# Intestinal Perforation as a Paradoxical Reaction to Tuberculosis

**DOI:** 10.7759/cureus.24077

**Published:** 2022-04-12

**Authors:** Mohamad B Alebaji

**Affiliations:** 1 Pediatric Medicine, Tawam Hospital, Abu Dhabi, ARE

**Keywords:** arab countries, uae, paradoxical reaction, tuberculosis, intestinal perforation

## Abstract

Paradoxical reactions (PR) to tuberculosis (TB) treatments are characterized by an initial improvement of the clinical symptoms followed by a clinical or radiological deterioration of existing TB lesions or by the development of new lesions. PR in the gastrointestinal system is a rare phenomenon. Moreover, intestinal perforation is an uncommon but potentially fatal complication of intestinal TB. We report the case of a 29-year-old female who presented with fever and abdominal pain that was associated with watery diarrhea. She was diagnosed as a case of intestinal TB. During her stay, she developed intestinal perforation following the initiation of anti-TB treatment. She was eventually managed as a case of intestinal perforation as a PR to TB.

## Introduction

For the last 25 years, tuberculosis (TB) has been deemed a global public health emergency and is the leading cause of death from infectious diseases among adults around the globe [1]. The World Health Organization (WHO) estimates suggest that 9.9 million individuals developed TB in 2020, and the majority of these people were residents of South-East Asia. However, only 6.4 million of these approximately 10 million people were given a diagnosis and an official notification about their illness. Each year, 1.3 million people die due to TB [2].

The sixth most frequent form of presentation of TB is gastrointestinal, representing 3-19% of extrapulmonary TB cases [3]. The gastrointestinal tract, spleen, liver, pancreas, lymph nodes, and peritoneum are involved in abdominal TB [4]. Obstruction, intestinal perforation, and fistula formation are some of the complications of gastrointestinal TB [5]. Intestinal perforation due to gastrointestinal TB occurs in 4-7.6% of cases, with an associated mortality rate of 30% [6].

The perforation may occur at the beginning of or before the initiation of treatment and, on average, it occurs nine months after the appearance of initial symptoms [6]. A paradoxical reaction (PR) is suspected in cases where intestinal perforation occurs during or after the treatment [7]. In a PR to TB treatment, clinical symptoms initially improve, but later a clinical or radiological deterioration of existing TB lesions or the onset of new lesions is observed [8,9]. Of note, 6-30% of patients receiving anti-tubercular therapy suffer from PR. We present a case of intestinal perforation occurring in a patient with normal immunity who was being treated for intestinal TB with anti-tubercular drugs.

## Case presentation

**Figure 1:**
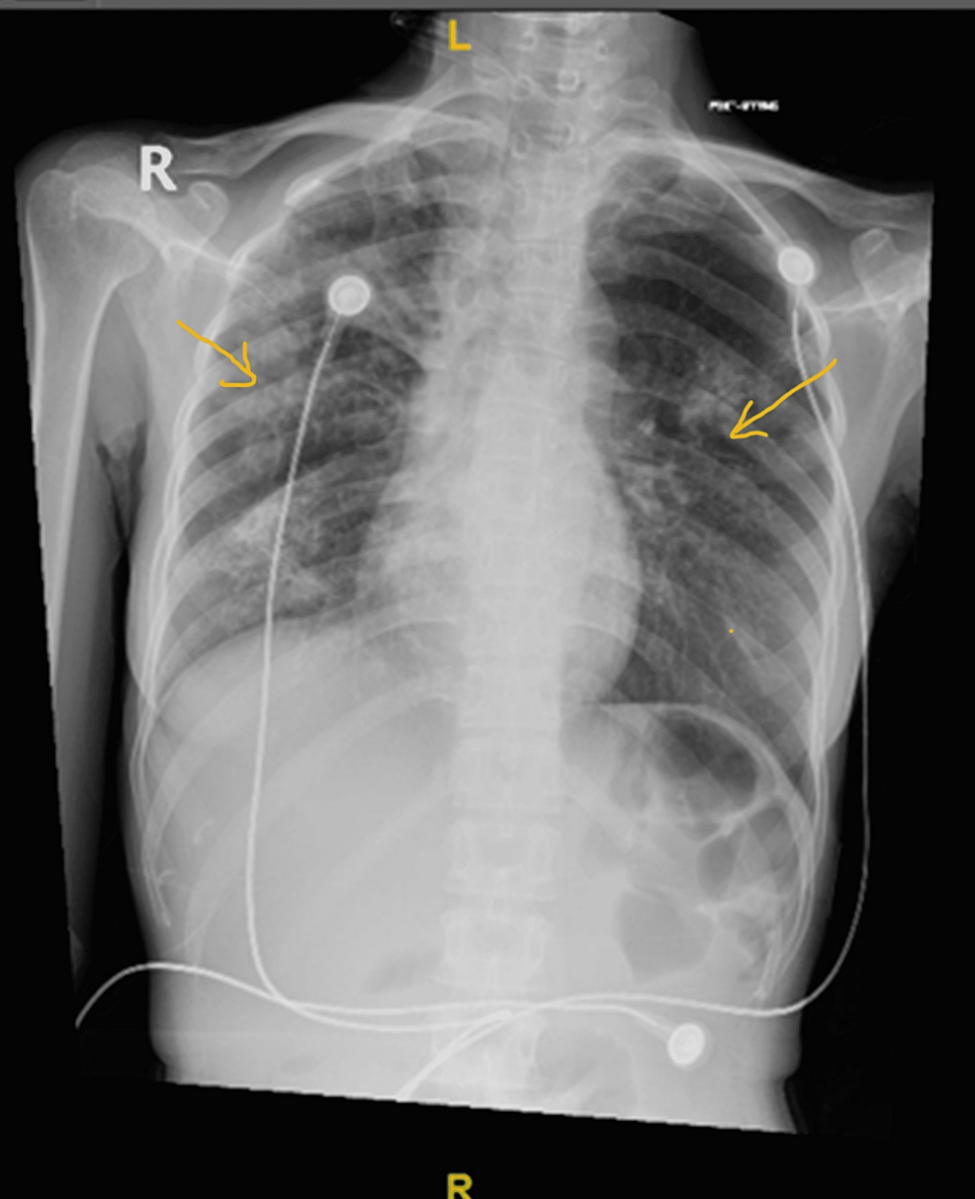
Bilateral inhomogeneous faint lung opacities, the early consolidative process likely of inflammatory origin

A 29-year-old female patient presented to the emergency department at our hospital; she had been unwell for the past one month with a fever and abdominal pain, which was associated with watery diarrhea. She had lost 15 kg of weight since the symptoms started. Her medical and surgical history was unremarkable, and she denied any recent travel. On physical examination, she was cachectic, dehydrated, tachycardia [heart rate (HR): 130 bpm], hypotensive (BP: 66/18 mmHg), with a temperature of 39 °C, and was maintaining a SpO_2_ of 92% on room air. A chest examination revealed scattered crepitation on auscultation; she denied coughing or shortness of breath. The rest of the systemic examination was unremarkable. Her initial blood test showed anemia with hemoglobin (Hb) of 8 mg/dl, but other baselines were within normal limits. Chest X-ray revealed bilateral inhomogeneous faint lung opacities (Figure 1). Three consecutive early-morning sputum samples were sent for acid-fast bacilli (AFB) culture, in which the first two samples were negative.

**Figure 2:**
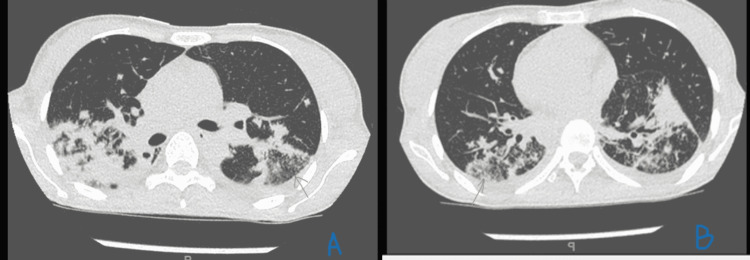
A. Right upper lobe apical and posterior segment consolidation with air bronchogram; shows tiny foci of parenchymal calcifications. B. Yet smaller lesions involving a superior segment of both lower lobes

High-resolution CT (HRCT) was done, which suggested active TB infection (Figure 2). Gamma interferon and TB polymerase chain reaction (PCR) were positive, and hence anti-TB medications were initiated with a once-daily regimen of isoniazid (400 mg), rifampin (600 mg), ethambutol (800 mg), and pyrazinamide (1500 mg).

**Figure 3:**
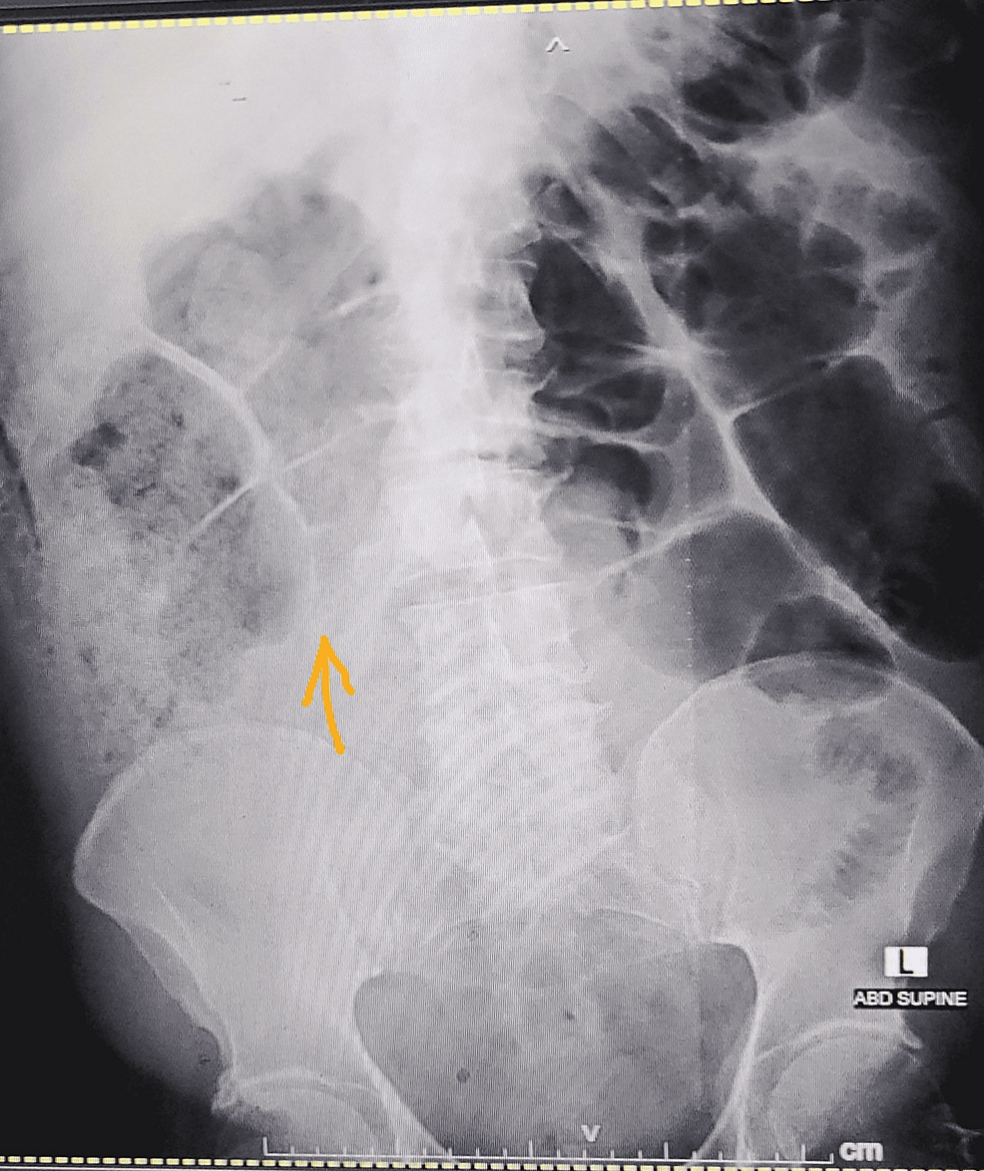
X-ray of the erect abdomen shows pneumoperitoneum

**Figure 4:**
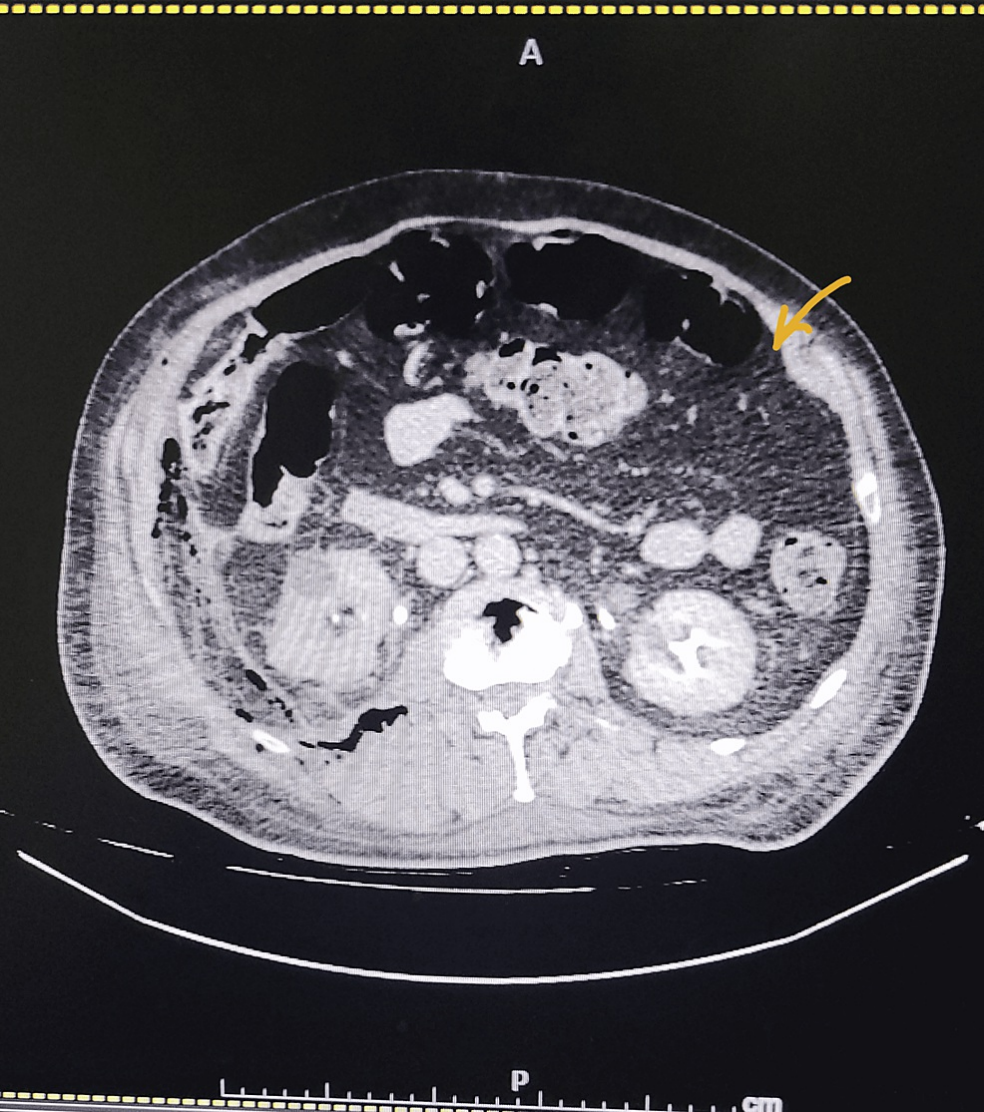
CT abdomen showing pneumoperitoneum CT: computed tomography

During her hospital stay, she had repeated hematemesis that caused a massive drop in Hb to 3 mg/dl. Oesophago-gastro-duodenoscopy (OGD) showed focal scarring of D1, suggestive of a healed duodenal ulcer. Subsequently, she had severe lower abdominal pain. Abdominal erect X-ray (Figure 3) and abdominal CT (Figure 4) were done, and both showed pneumoperitoneum. The surgeon was consulted, and she underwent laparoscopic exploration for intestinal perforation, which was converted to an open laparotomy for perforated viscous. A small perforation was seen 150 cm from the ileocecal valve, and during manipulation and adhesiolysis, a second perforation occurred near the first one.

**Figure 5:**
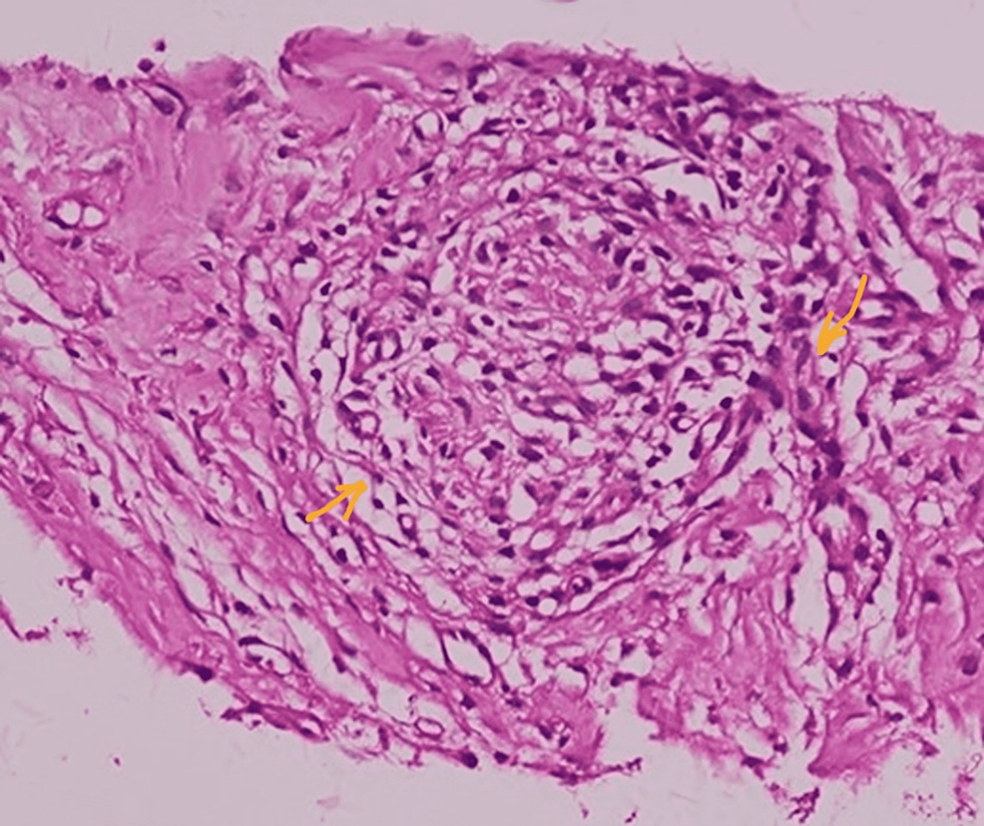
Histopathological presentation of the specimen sections revealed appendiceal tissue showing chronic inflammatory cellular reaction and few granulomas in serosa and fatty tissue. Lymphoid follicular hyperplasia is observed

Biopsies from perforated viscus showed chronic granulomatous appendicitis suggestive of TB (Figure 5). Only one month after admission, the third AFB sample returned positive. Anti-TB medications were continued for another nine months, and the patient's condition improved; she was sent home with oral medications. The patient has been followed up for one year after the surgery and has remained asymptomatic. She has provided informed consent for the use of her medical information in this case report.

## Discussion

The pleura, lymph nodes, central nervous system, bones, and abdomen can be affected by extrapulmonary TB. Gastrointestinal TB accounts for 17% of all extrapulmonary cases and is the most common presentation of abdominal TB [5]. Intestinal perforation occurs in an estimated 1-15% of all intestinal TB patients [6].

PR is characterized by a radiological or clinical worsening of pre-existing lesions or the development of new lesions in patients who showed an initial improvement when receiving anti-TB treatment [8]. Typically, it is a transient, mild, and self-limiting phenomenon, but higher morbidity and mortality have been reported when the central nervous system is involved [10]. In increasing order of frequency, PR has been reported in patients with TB affecting the abdomen, lymph nodes, skin/soft tissues, respiratory system, and nervous system. One report suggests that new lesions at other sites occur in approximately 25% of the patients while worsening of primary lesions occurs in approximately 75% of the patients [9].

In previous studies, PR has been typically reported to develop within one to six months after starting anti-TB therapy [11]. Moreover, poor nutritional status has been cited as a possible factor contributing to intestinal perforation [12]. Intestinal perforation is an uncommon complication of intestinal TB that occurs due to the formation of adhesions within the surrounding tissues following a reactive thickening of the peritoneum.

A complete understanding of the pathophysiological mechanism of the PR has not yet been developed. Delayed hypersensitivity of the host is strengthened by the heightened exposure to mycobacterial antigens released from the bacilli that were killed by effective anti-tubercular therapy. PR is related to a direct effect of the host over bacterial products and is believed to be a delayed hypersensitivity reaction to bacterial antigens [10]. Tumor necrosis factor-alpha (TNF-α) is believed to play an important role as PR cases have reportedly been associated with the cessation of biological anti-TNF-α therapies in patients with Crohn’s disease [13].

## Conclusions

We described the case of a patient who experienced intestinal perforation as a PR to TB. In patients with intestinal TB, with the reappearance of symptoms or after an initial improvement with anti-TB therapy following new manifestations, a gastrointestinal PR should be suspected. The most serious manifestation of PR is intestinal perforation.
