# Correction: Intestinal Perforation as a Paradoxical Reaction to Tuberculosis

**DOI:** 10.7759/cureus.c65

**Published:** 2022-06-10

**Authors:** Mohamad B Alebaji

**Affiliations:** 1 Pediatric Medicine, Tawam Hospital, Abu Dhabi, ARE

Abdelrahman I. Omara has been removed as second author from this case report per their request as it was erroneously included by the submitting author due to a misunderstanding prior to submission of the manuscript to Cureus.

